# Baicalin improves the functions of granulosa cells and the ovary in aged mice through the mTOR signaling pathway

**DOI:** 10.1186/s13048-022-00965-7

**Published:** 2022-03-17

**Authors:** Huiying Fan, Jiahuan He, Yucheng Bai, Qina He, Tongwei Zhang, Junya Zhang, Guang Yang, Ziwen Xu, Jingyi Hu, Guidong Yao

**Affiliations:** 1 Center for Reproductive Medicine, the First Affiliated Hospital of Zhengzhou University, Zhengzhou, China; 2 Henan Key Laboratory of Reproduction and Genetics, the First Affiliated Hospital of Zhengzhou University, Zhengzhou, 450052 China

**Keywords:** Baicalin, Follicular development, Granulosa cell, Ovarian function, mTOR pathway

## Abstract

**Background:**

The mammalian follicle is the basic functional unit of the ovary, and its normal development is required to obtaining oocytes capable of fertilization. As women get older or decline in ovarian function due to certain pathological factors, the growth and development of follicles becomes abnormal, which ultimately leads to infertility and other related female diseases. Kuntai capsules are currently used in clinical practice to improve ovarian function, and they contain the natural compound Baicalin, which is a natural compound with important biological activities. At present, the role and mechanism of Baicalin in the development of ovarian follicles is unclear.

**Methods:**

Human primary granulosa cells collected from follicular fluid, and then cultured and treated with Baicalin or its normal control, assessed for viability, subjected to RT-PCR, western blotting, flow cytometry, and hormone analyses. The estrus cycle and oocytes of CD-1 mice were studied after Baicalin administration and compared with controls. Ovaries were collected from the mice and subjected to hematoxylin-eosin staining and immunohistochemistry analysis.

**Results:**

We showed that Baicalin had a dose-dependent effect on granulosa cells cultured in vitro. A low concentration of Baicalin (for example, 10 μM) helped to maintain the viability of granulosa cells; however, at a concentration exceeding 50 μM, it exerted a toxic effect. A low concentration significantly improved the viability of granulosa cells and inhibited cell apoptosis, which may be related to the resultant upregulation of Bcl-2 expression and downregulation of Bax and Caspase 3. By constructing a hydrogen peroxide-induced cell oxidative stress damage model, we found that Baicalin reversed the cell damage caused by hydrogen peroxide. In addition, Baicalin increased the secretion of estradiol and progesterone by upregulating P450arom and stAR. The results of the in vivo experiment showed that the intragastric administration of Baicalin to aged mice improved the estrous cycle and oocyte quality. Furthermore, we observed that Baicalin enhanced the viability of granulosa cells through the mTOR pathway, which in turn improve ovarian function.

**Conclusion:**

These results indicate that Baicalin could improve the viability of ovarian granulosa cells and the secretion of steroid hormones and thus could help to improve degenerating ovarian function and delay ovarian aging.

**Supplementary Information:**

The online version contains supplementary material available at 10.1186/s13048-022-00965-7.

## Introduction

Mammalian follicles are the basic functional units of the ovary and are composed of oocytes surrounded by granulosa cells. Granulosa cells undergo proliferation, differentiation, and apoptosis in response to different hormones and cytokines during follicle growth, regulating their development and oocyte maturation. The normal function of granulosa cells is closely related to the quality of oocytes and ovarian function, and their dysfunction is associated with diseases related to abnormal ovarian and follicular development [1].

Female reproductive aging is accompanied by a progressive decline in the number of follicles and quality of oocytes, leading to menstrual disorders and even menopause, which results in the loss of ovarian function and infertility. Abnormal follicular development and disordered estrogen secretion can induce relevant diseases and even affect female fertility. The gradual decrease in the follicle population with age in mice may be related to the increased expression of systemic pro-inflammatory and inflammasome-related genes [2]. In addition, abnormal follicular development under certain pathological conditions and ovarian follicular developmental arrest or premature follicular depletion due to damage from drug stimulation are also important causes of infertility [3].

Kuntai capsules contain a Chinese herbal formula that has been widely used in the clinical treatment of menopausal syndromes. Studies have confirmed that Kuntai capsules have a protective effect on ovarian reserves and fertility related to the PI3K/AKT/mTOR signaling pathway [4]. Clinical studies have shown that Kuntai capsules combined with hormone therapy are more effective and safer than hormone therapy alone for premature ovarian failure [5]. Animal experiments have also confirmed that Kuntai capsules can regulate the estrous cycle, promote hormone secretion, improve fertility, reduce the incidence of follicular atresia, improve the ultrastructures of ovaries, and reduce cell apoptosis in mice with impaired ovarian function [7]. Moreover, Kuntai capsules increase the number of ovarian follicles, inhibit oocyte apoptosis, and help to maintain ovarian function [8].

Baicalin is the main component of *Scutellaria baicalensis* and an important active component in Kuntai capsules. It was first isolated from *S. baicalensis* in 1889 and has attracted increasing attention from researchers because of its wide range of pharmacological effects, low toxicity, and abundance in the roots of *Scutellaria baicalensis* [9]. As one of the important flavonoid compounds, Baicalin has a wide range of pharmacological effects, such as anti-tumor, anti-angiogenesis, liver protection, anti-oxidation, anti-convulsion, anti-bacterial, and anti-viral effects [10]. Baicalin can shorten the intervals between estrus and play an important role in improving ovarian function and follicular development in polycystic ovary syndrome rats [11]. However, the role of Baicalin in regulating the development of ovarian follicles remains unclear.

The mammalian target of rapamycin (mTOR) plays an important regulatory role in the process of cell metabolism, proliferation, and differentiation and is expressed in granulosa cells and oocytes at all stages of follicular development [12]. As a key pathway for ovarian function, the mTOR signaling pathway has an important effect on the growth and development of primordial follicles, proliferation and differentiation of granulosa cells, and meiosis of oocytes [13]. Oocyte-specific mTOR knockouts can reduce oocyte quality and the fertility of female mice, and hinder the first meiotic process in oocytes [12]. Studies have confirmed that rapamycin can prevent the activation of primordial follicles induced by cyclophosphamide through the PI3K/AKT/mTOR signaling pathway, resulting in a certain protective effect on the ovarian follicle reserve [14]. However, further experimental verification is required to determine if Baicalin regulates the development of ovarian follicles through the mTOR pathway and its mechanisms of action.

In this study, we analyzed the effects of Baicalin on in vitro culture of human primary granulosa cells and identified its potential mechanisms of action. Further, in vivo experiments were conducted to explore the active effects of Baicalin on the ovarian functions of aged mice.

## Materials and methods

### Ethical approval and consent to participate

This study has been approved by the Biomedical Ethics Committee of the First Affiliated Hospital of Zhengzhou University, and all patients signed an informed consent form.

### Animals

All CD-1 mice were purchased from Beijing Vital River Laboratory Animal Technology Co. Ltd. (Vital River, Beijing, China) and were maintained under specific pathogen-free conditions with a standard light–dark cycle and free access to food and water. The use of experimental animals in this study was approved by the Biomedical Ethics Committee of the First Affiliated Hospital of Zhengzhou University.

### Cell culture

Human follicular fluid was collected from patients who were less than 35 years old with a normal ovulation cycle and who had no history of ovarian surgery, and infertile women whose infertility was caused by tubal factors or male factors [1]. In order to eliminate individual differences, each experiment collects 5 to 6 patients’ follicular fluid and mixes them, and primary granulosa cells were collected from the follicular fluid for subsequent experiments, and the cells were purified using density gradient centrifugation [16].

The obtained human primary granulosa cells and KGN granulosa cell line (gifted from Prof. Fei Sun in University of Nantong in China) were cultured in Dulbecco’s modified Eagle medium (DMEM)/F12 medium (1:1, Thermo Fisher, Waltham, USA) supplemented with 10% fetal bovine serum (FBS; HyClone, Logan, UT, USA) and 1% penicillin/streptomycin (HyClone) at 37 °C, 5% CO_2_, and saturated humidity. Before the cells were subjected to the treatments, the DMEM/F12 medium containing 0.5% FBS and 1% penicillin/streptomycin was used as a starvation culture medium. Baicalin (Must Bio-Technology, Chengdu, People’s Republic of China) was prepared in a 50 μM storage solution with dimethyl sulfoxide (Thermo Fisher Scientific, Waltham, MA, USA) and then diluted to the required working concentration (1 μM, 5 μM,10 μM, 25 μM, 50 μM, 100 μM, 150 μM, and 200 μM) with the starvation culture medium during use.

### Cell viability analysis

Our pre-experimental studies have found that the results obtained by human primary granulosa cells seeded in 24-well plates for cell viability analysis are more stable and reliable than those obtained when seeded in 96-well plates. Therefore, KGN cell lines were seeded in 96-well plate at the density of 2–3 × 10^3^ cells/well, and the primary human granulosa cells were seeded in 24-well plate at the density of 3–4 × 10^4^ cells/well, respectively, and cultured for 24 h. The cells were cultured in the starvation medium for 24 h, and then treated with Baicalin (0–200 μM). Before testing the cell viability, the medium in the culture plates was replaced with DMEM/F12 medium containing 10% Cell Counting Kit (CCK)-8 reagent (Dojindo Molecular Technologies, Tokyo, Japan), and then the cells were then incubated for 2 h in an incubator at 37 °C and 5% CO_2_. The optical density (OD) values were measured at 450 nm using a microplate reader (Thermo Fisher Scientific). To establish the hydrogen peroxide-induced cell oxidative stress damage model, granulosa cells were pretreated with Baicalin for 72 h, followed by incubation with 1.5 mM of 3% hydrogen peroxide (MilliporeSigma, Burlington, MA, USA) in starvation culture medium for 3 h. The CCK-8 kit was then used to detect cell viability according to the manufacturer’s instructions.

In the cell viability analysis using MHY1485 (mTOR activator, Calbiochem, Sigma-Aldrich, St. Louis, MO, USA) and HY-112914 (mTOR inhibitor, Calbiochem), the primary granulosa cells were first evenly seeded onto 24-well plates and conventionally cultured for 24 h. Subsequently, the cells were incubated in the starvation culture medium for 24 h and 10 μM MHY1485 or HY-112914 was added as the pretreatment for 30 min. Specific concentrations of Baicalin were then added, and the treatment continued for 72 h; finally, a CCK-8 kit was used to measure cell viability. All experiments were repeated three times independently with six replicate wells for each experiment.

### RNA extraction and real-time PCR

The culture medium was removed following the treatment of the granulosa cells. After the granulosa cells were washed with pre-cooled phosphate-buffered saline (PBS), RNA extraction was performed using TRIzol Reagent (Invitrogen, Thermo Fisher Scientific) according to the manufacturer’s instructions. RNA quality and concentration at OD_260/280_ nm were detected using NanoDrop-2000 (Thermo Fisher Scientific). After reverse transcription of the total RNA into cDNA using an RNA reverse transcription kit (iScript Reverse Transcription Supermix for RT-qPCR, Bio-Rad, Hercules, CA, USA), real-time PCR was performed using an iTaq Universal SYBR Green Supermix (Bio-Rad) kit and 7500 Fast Real-Time PCR (Thermo Fisher). The above experimental techniques were carried out in accordance with the manufacturer’s instructions. The relative gene expression levels were calculated using the 2^-ΔΔCt^ method with *GAPDH* as an internal reference gene. The primer sequences used are shown in Supplementary Table 1.

### Western blotting

The human primary granulosa cells were seeded in 6-well plates at the density of 2 × 10^6^ cells/well, conventionally cultured for 24 h, and then starved in the starvation culture medium for 24 h. Subsequently, the cells were treated with the corresponding concentrations of Baicalin for 48 h, and the medium was then removed. After washing the cells with pre-cooled PBS, a protein extraction kit (Sangon Biotech, Shanghai, People’s Republic of China) was used to lyse the cells, followed by quantification of the protein concentration using a rapid protein quantification kit (Thermo Fisher Scientific). Sodium dodecyl sulfate–polyacrylamide gel electrophoresis was performed to separate the denatured proteins, which were then electrotransferred to a polyvinylidene fluoride membrane. Subsequently, the membrane was placed in 5% skim milk supplemented with Tris-buffered saline with 0.1% Tween 20 (TBST; Solarbio, Beijing, People’s Republic of China) for 1 h. The membrane was then incubated with primary antibodies overnight at 4 °C. The next day, the membrane was washed with TBST three times for 5 min each, followed by incubation with the secondary antibodies coupled with horseradish peroxidase (HRP) for 1 h and then washed with TBST for 30 min at 22–26 °C. The Protein Band Enhanced Chemiluminescent Detection System (Bio-Rad) and ChemiDoc MP Imaging System (Bio-Rad) were used to image the chemiluminescent spots.

The following primary antibodies were used: Bax (1:2000; Proteintech, Wuhan, People’s Republic of China), Bcl-2 (1:5000; Abcam), Caspase 3 (1:2000; Abcam), p-mTOR (1:2000; Abcam), GAPDH (1:5000; Abcam), and α-tubulin (1:2000; Abcam). The following secondary antibodies were used: mouse IgG (1:5000; Abcam) and rabbit IgG (1:5000; Abcam).

### Flow cytometry analysis

The human primary granulosa cells were seeded in 12-well plates at the density of 3–4 × 10^5^cells/well, conventionally cultured for 24 h, and then starved in the starvation culture medium for 24 h. The adherent granulosa cells were collected by trypsin digestion after treatment with 10 μM Baicalin for 72 h, according to the instructions of an Annexin V-FITC/PI Apoptosis Detection Kit (KeyGen Biotech, Nanjing, People’s Republic of China). Briefly, the cells were collected using a trypsin digestion solution without EDTA, washed twice with pre-cooled PBS, and centrifuged at 2000 rpm for 5 min. Subsequently, the cell pellet was suspended in 500 μL binding buffer, and 5 μL Annexin-FITC was added and mixed well. Then, 5 μL of propidium iodide was added and mixed well, and the mixture was kept in the dark at 22–25 °C for 5–15 min, followed by cell apoptosis detection using BD C6 Flow Cytometry (BD Biosciences, San Jose, CA, USA) within 1 h.

### Hormone analysis

The primary granulosa cells were seeded onto 6-well plates at a density of 1 × 10^6^ cells per well, conventionally cultured for 24 h, starved in the starvation culture medium for 24 h, and then treated with the corresponding concentrations of Baicalin with 10^− 7^ M testosterone (Solarbio) added simultaneously to the medium as the substrate for estradiol (E_2_) production. After 48 h of treatment, the cell culture supernatant was collected, and the concentrations of estradiol and progesterone were analyzed using a chemiluminescent immunoassay kit (Roche Diagnostics, Rotkreuz, Switzerland) and cobas 6000 analyzer series (Roche Diagnostics) according to the kit manufacturer’s instructions.

### Mouse estrous cycle detection

Ten-month-old CD1 mice were randomly divided into control and Baicalin treatment groups. The mice in the Baicalin group were intragastrically administered 100 mg/kg Baicalin every other day, whereas the mice in the control group were intragastrically administered an equal volume of normal saline every other day. The estrous cycle was monitored after 60 days of intragastric administration. A small amount of secretion was acquired from the lateral vaginal wall of the female mice through gentle scratching between 8:00 am and 9:00 am every morning using a small sterile cotton swab wetted with a small amount of PBS and then evenly coated on a glass slide, fixed with 95% ethanol, stained with hematoxylin-eosin (HE), and photographed under a microscope to observe the changes and differences in the estrous cycles of the female mice. The phase of the estrous cycle was determined based on the ratio of the nucleated and keratinized epithelial cells and leukocytes [17].

Diestrus was identified when the cells were predominantly leukocytes with few nucleated keratinocytes, which gradually increased in number. Proestrus was characterized by nucleated epithelial cells, which gradually decreased in number, and leukocytes, which gradually disappeared. Estrus was identified by nucleated keratinized epithelial cells without a predominance of leukocytes. Finally, metestrus was characterized by keratinized epithelial cells, which gradually decreased in number, and an increasing number of leukocytes.

### Cumulus–oocyte complex (COC) acquisition

After 90 days of intragastric administration, the mice in the control and the Baicalin treatment groups were intraperitoneally injected with 10 U PMSG (Solarbio) and with 10 U human chorionic gonadotropin (hCG; Solarbio) 48 h later. The female mice were euthanized by cervical dislocation 16 h after injection with hCG. COCs were obtained from the ampulla of the fallopian tube and placed in G-MOPS Plus (Vitrolife AB, Göteborg, Sweden) under an inverted microscope (TE2000-U, Nikon, Japan) for photography and observation. The cumulus cells of the COCs were removed by repetitive aspiration with a fine needle (Sunlight Medical, Jacksonville, FL, USA) with hyaluronidase (Vitrolife AB) digestion. The oocytes stripped of granulosa cells were photographed and observed under an inverted microscope. Observed under a microscope, the cytoplasm of normal oocytes is shiny, and the perivitelline space is normal; the cytoplasm of degenerated oocytes is dull, and the cytoplasm collapses and fills the entire zona pellucida; while the cytoplasm of pycnotic oocytes shrinks and becomes black and the perivitelline space has increased abnormally.

### HE staining and immunohistochemistry

After the mice were euthanized by cervical dislocation, the ovaries were stripped and fixed in 4% paraformaldehyde (Solarbio), followed by routine embedding, and the paraffin was then successively sliced at a thickness of 5 μm and placed on glass slides. Subsequently, the wax blocks were deparaffinized and hydrated with xylene and ethanol, followed by HE staining and immunohistochemical analysis.

The slides for the immunohistochemical analysis were first treated with sodium citrate antigen retrieval solution (Servicebio, Wuhan, People’s Republic of China) for antigen retrieval, and then, 3% hydrogen peroxide solution was added to block endogenous peroxidase. After blocking with bovine serum albumin (Servicebio) at 22–25 °C for 30 min, the primary antibody p-mTOR (1:2000; Abcam) was added, followed by overnight incubation at 4 °C; the serum was used as a negative control instead of the primary antibody. The slides were washed after incubation with the primary antibody and then incubated with the HRP-labeled mouse IgG secondary antibody (1:5000; Abcam) for 50 min. Finally, 3,3′-diaminobenzidine (Servicebio) staining was performed until a brownish yellow color was observed. The positive control samples were obtained by treating the ovaries of day 4 newborn mice with 200 μM phosphatidic acid (PA) for 24 h in vitro, and immunohistochemical staining for p-mTOR was performed.

### Statistical analysis

When processing the resultant data from this experiment, three or more values were expressed as the mean ± standard error of the mean, and all data were analyzed using SPSS (version 17.021.0) statistical software (IBM, Armonk, NY, USA). Moreover, *t*-tests were applied for statistical analysis and chi-squared tests were used for rate statistics. *P* < 0.05 was used to indicate a significant difference.

## Results

### Baicalin had a dose-dependent effect on the cultured granulosa cells in vitro

**Fig. 1:**
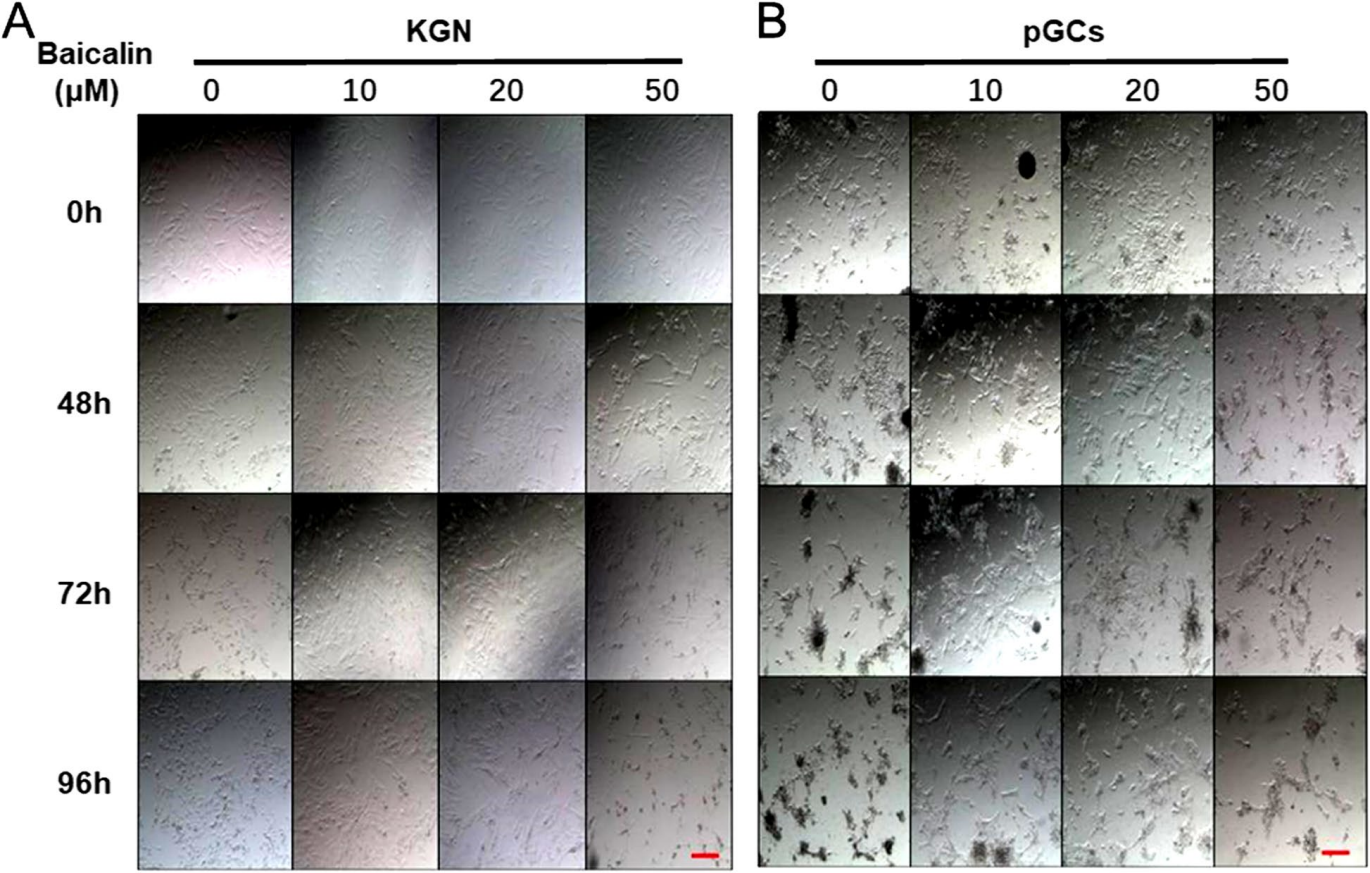
Effects of different concentrations of Baicalin on cultured KGN and primary granulosa cells in vitro. The KGN and primary granulosa cells were planted in 24-well plates, and conventionally cultured in DMEM/F12 medium supplemented with 10% FBS and 1% antibiotics/antimycotics for 24 h, and then the medium was replaced by DMEM/F12 containing 0.5% FBS and 1% antibiotics/antimycotics for the starvation culture. After 24 h in the starvation culture, different concentrations of Baicalin (0, 10, 20, and 50 μM) were added. The processing start time was set to 0 h, and then observations were made, and photographs taken after 48, 72, and 96 h. The left picture shows the KGN granulosa cell line (**A**), and the right picture shows the primary granulosa cells (**B**). Scale bar = 50 μm

The primary granulosa cells were first treated with different concentrations of Baicalin, and the results showed that the different concentrations had different effects on the KGN granulosa cells and primary granulosa cells. In the control group, the adherent cells become fewer and the morphology of the cells was obviously abnormal with reduced volume and shorter protrusions after the in vitro culture for 96 h, whereas the granulosa cells in the low-concentration (such as 10 and 20 μM) Baicalin treatment group still had good cell morphology and proliferation ability. However, when the Baicalin concentration reached 50 μM, the cell status was not much different from that of the control group (Fig. 1A & B). Higher concentrations of Baicalin (such as 100 and 200 μM) had a toxic effect on the granulosa cells, as the cells underwent obvious death and shrinkage after 24 h of treatment (Supplementary Fig. S1). These results indicated that Baicalin had a dose-dependent effect on human granulosa cells cultured in vitro. As the physiological concentrations of Baicalin in the body are generally low, this study focused on the effects of the low concentrations of the Baicalin on the granulosa cells.

### Effects of Baicalin on the proliferation and apoptosis of granulosa cells cultured in vitro

**Fig. 2:**
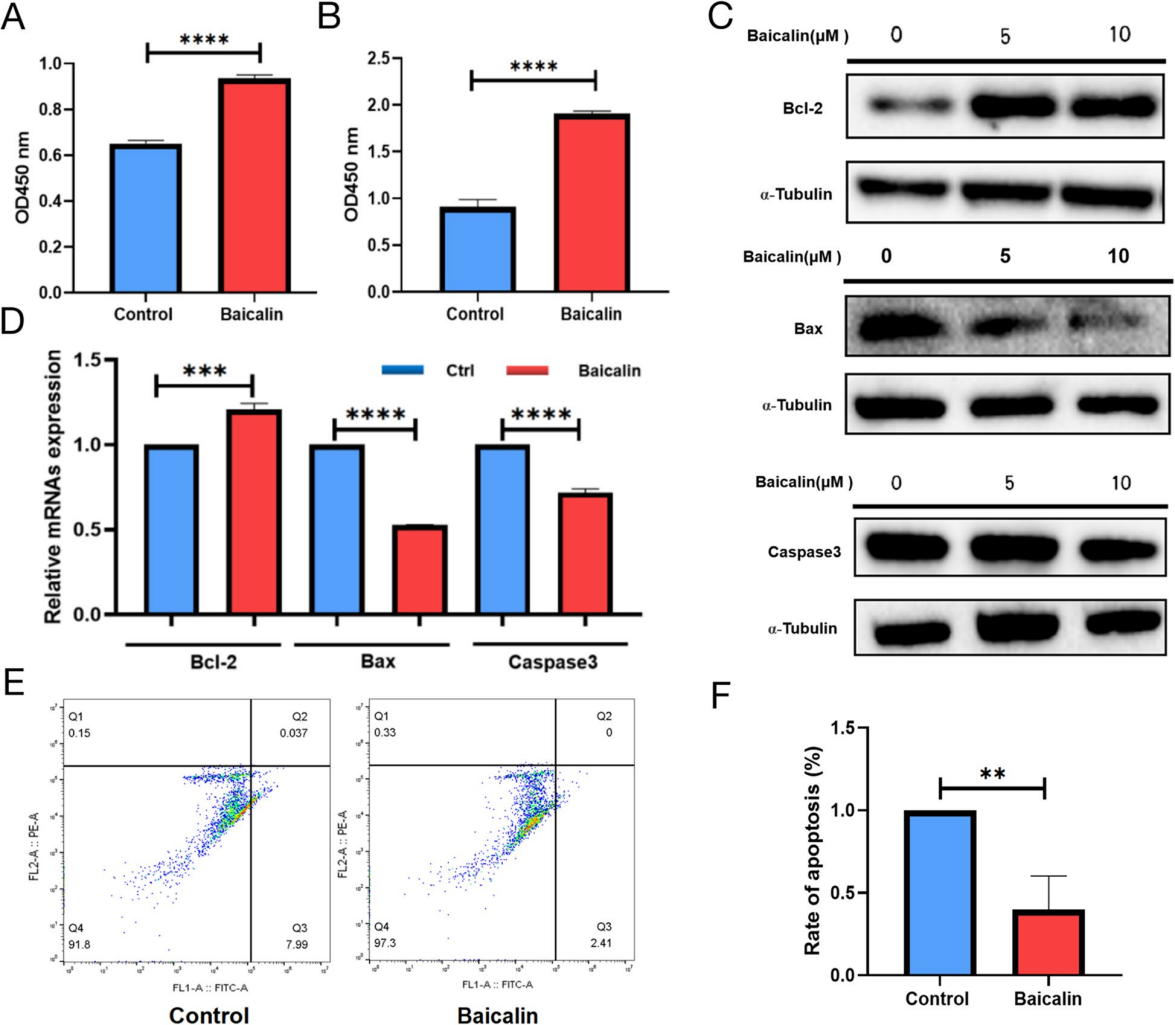
Effects of Baicalin on the proliferation and apoptosis of granulosa cells. After conventional culture, the granulosa cells were starved for 24 h, and then Baicalin (10 μM) was added. The CCK-8 kit was used to detect the OD450 values of the KGN cells (**A**) and primary granulosa cells (**B**) 72 h after treatment. Cell viability was compared between the different treatment groups. The human primary granulosa cell proteins were extracted 48 h after Baicalin treatment, and the protein expression levels of Bcl-2, Bax, and Caspase 3 were detected using western blotting (**C**). Total RNA of the human primary granulosa cells treated with Baicalin for 48 h was extracted using TRIzol, and the mRNA expression levels of Bcl-2, Bax, and Caspase 3 were detected using real-time PCR (**D**). Flow cytometry was used to detect the effects of Baicalin on the apoptosis of human primary granulosa cells (**E** & **F**). ** *P* < 0.01, *** *P* < 0.001, and **** *P* < 0.0001

Baicalin exerted a dose-dependent effect on the granulosa cells cultured in vitro, and low concentrations of Baicalin exerted beneficial effects on the in vitro cultures. We thus speculated that Baicalin is involved in the survival of the granulosa cells in vitro. The results showed that the viability of the KGN cells and primary granulosa cells treated with Baicalin for 72 h was significantly higher than that of the control group (*P* < 0.0001; Fig. 2 A and B). By analyzing the changes in the protein expression levels of *BCL-2*, *Bax* and *Caspase 3*, the results showed that Baicalin upregulated the protein levels of *BCL-2* and down-regulated the protein expression levels of *Bax* and *Caspase 3* (Fig. 2C). The results of the real-time fluorescent quantitative PCR showed that the mRNA expression levels of the Bcl-2 increased significantly after the Baicalin treatment (*P* < 0.001), whereas those of the Bax and Caspase 3 decreased significantly (*P* < 0.0001; Fig. 2D). In addition, the results of the flow cytometry analysis showed that the apoptosis ratio of the granulosa cells after the Baicalin treatment was significantly lower than that of the control group (*P* < 0.01; Fig. 2E and F).

### Baicalin partially reversed the oxidative stress damage to the granulosa cells induced by hydrogen peroxide

**Fig. 3:**
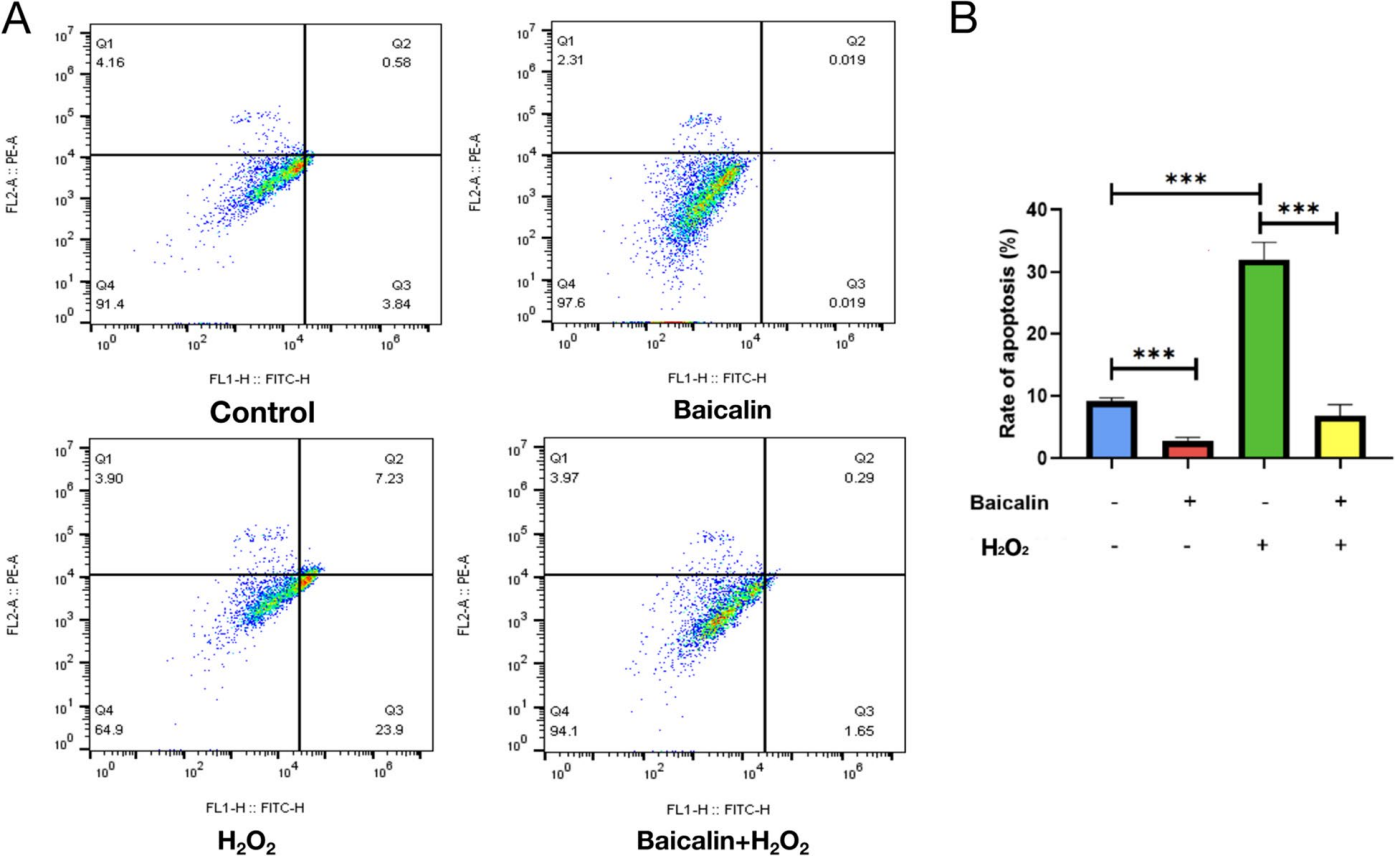
Baicalin could partially reverse the oxidative stress damage of granulosa cells induced by hydrogen peroxide. After conventional culture, primary granulosa cells were starved for 24 h, followed by incubation with Baicalin for 72 h and treatment with 1.5 mM hydrogen peroxide for 3 h at the same time. The apoptosis rate (**A** & **B**) was detected using a flow cytometry apoptosis kit. *** *P* < 0.001

To further explore the effects of Baicalin on the granulosa cells cultured in vitro, we established a hydrogen peroxide-induced cell oxidative stress damage model. The primary granulosa cells were conventionally cultured and then treated with Baicalin, followed by a hydrogen peroxide stress treatment. The results of the flow cytometry analysis showed that the Baicalin treatment significantly inhibited the apoptosis of granulosa cells induced by hydrogen peroxide (*P* < 0.0001; Fig. 3A and B). These results indicated that Baicalin decreased the influence of the oxidative stress on the granulosa cell viability and reduced cell apoptosis.

### Baicalin promoted steroid hormone production in granulosa cells

**Fig. 4:**
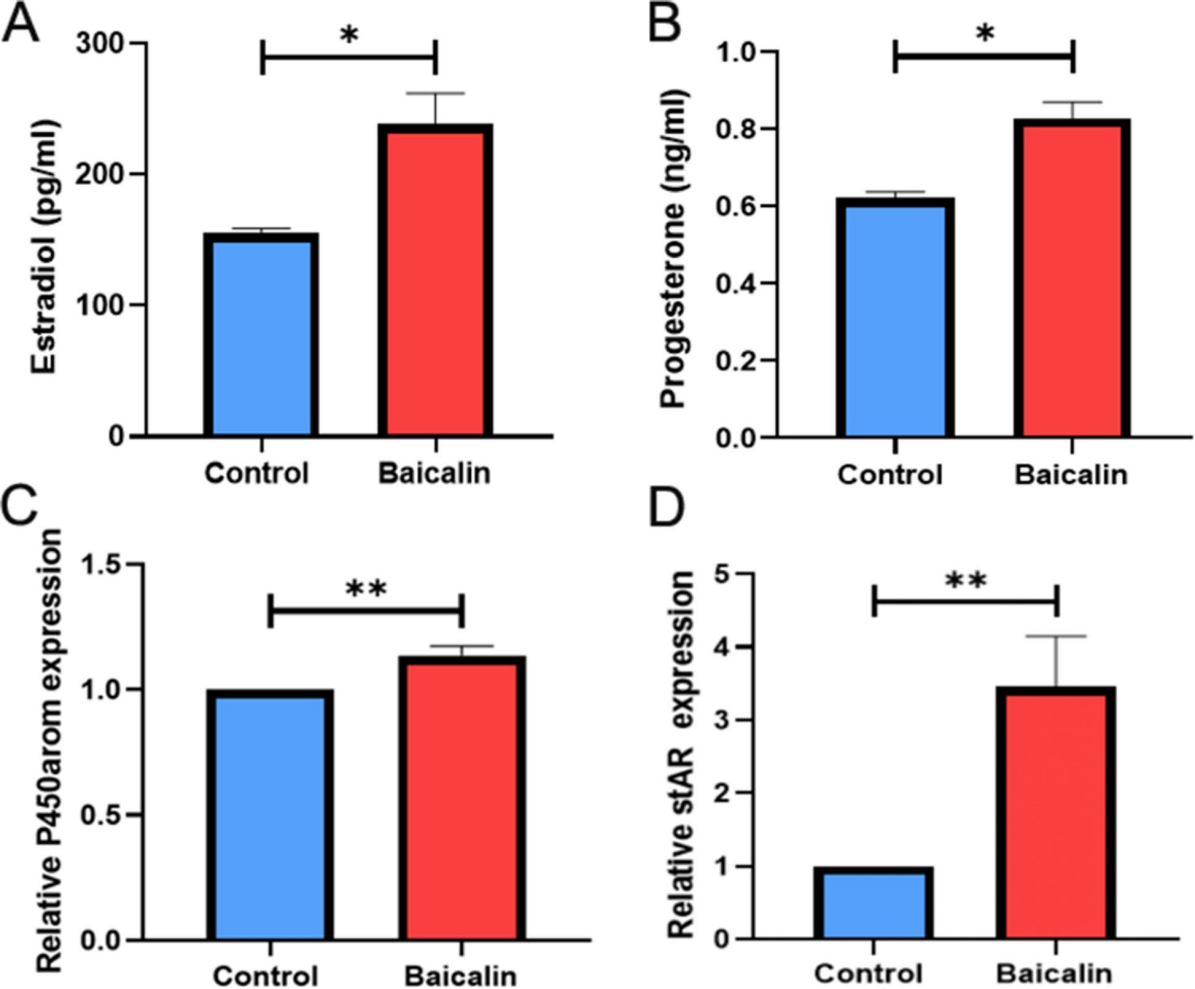
Effects of Baicalin on the steroid hormone production ability of ovarian granulosa cells in vitro. Primary granulosa cells were purified and planted in 6-well plates, followed by conventional culture for 24 h, and then starved for 24 h. After treatment with Baicalin (10 μM) for 48 h, the cell culture medium was collected and centrifuged to obtain the supernatant, followed by detection of the levels of estradiol (**A**) and progesterone (**B**) using a chemiluminescent immunoassay kit. The total RNA of the granulosa cells was extracted using TRIzol, and the mRNA expression levels of *P450arom* (**C**) and *stAR* (**D**) were detected using real-time PCR. * *P* < 0.05, and ** *P* < 0.01

To investigate whether the Baicalin treatment could affect the steroid hormone production in granulosa cells, we further explored the production of estrogen and progesterone in the granulosa cells after treatment with Baicalin. The amount of estradiol and progesterone in the supernatant was determined by collecting the culture supernatant of the primary granulosa cells treated with Baicalin. The results showed that the levels of estradiol and progesterone in the cell supernatant of the Baicalin treatment group were significantly higher than those of the control group (*P* < 0.05; Fig. 4A and B). Moreover, Baicalin significantly increased the mRNA expression levels of *P450arom* and *stAR*, which are closely related to the production of steroid hormones after Baicalin treatment (*P* < 0.01; Fig. 4C and D).

### Baicalin improved the estrous cycle in aged mice and improved egg quality

**Fig. 5:**
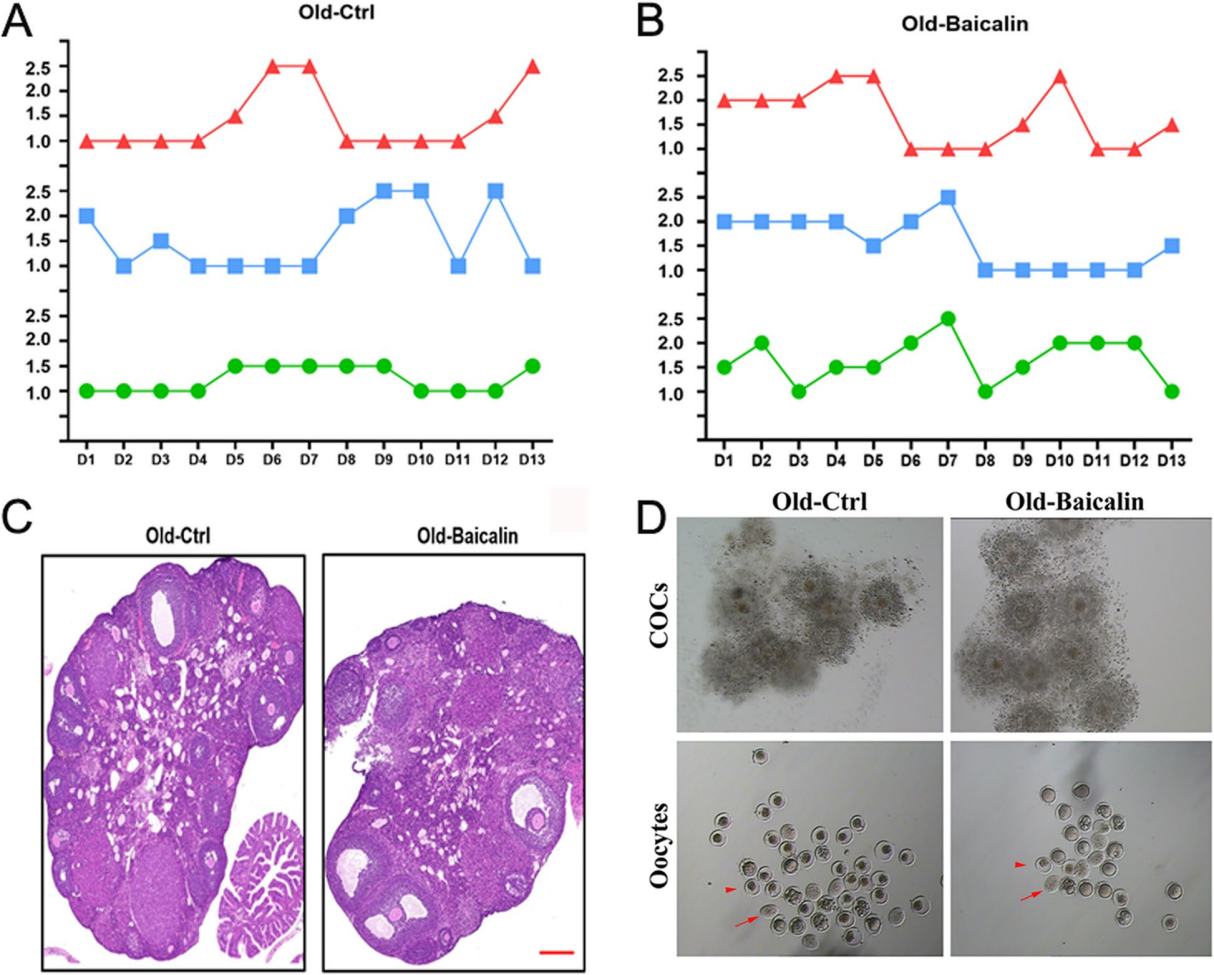
Effects of Baicalin on the estrous cycle and egg quality of aged female mice. Different treatments were given to 10-month-old female CD1 mice: intragastric administration with Baicalin (100 mg/kg) every other day in the aged Baicalin treatment group (Old-Baicalin), and intragastric administration with saline every other day in the aged control group (Old-Ctrl). After 60 days of intragastric administration, vaginal smears were obtained daily for HE staining and the phase of the estrous cycle for the mice was determined according to the ratio of keratinocytes and leukocytes. In the figure, along the y-axis, 1.0 represents diestrus, 1.5 represents proestrus, 2.0 represents estrus, and 2.5 represents metestrus. Line charts of the estrous cycles for the mice in the aged control group (**A**) and the aged Baicalin treatment group (**B**) were drawn. The lines with different colors in the two figures (**A** & **B**) represent the estrus situation of the three different mice in the two treatment groups. After 90 days of intragastric administration, the ovaries of the two groups were routinely fixed, embedded, and then sliced for HE staining to observe follicle development (**C**). Scale bar = 200 μm. Superovulation was performed in the mice after intragastric administration, followed by the acquisition of the COCs from the ampulla of the fallopian tube, and the morphological changes of the COCs in the control and Baicalin treatment groups were photographed and compared (above), as well as the oocytes stripped with the cumulus cells by hyaluronidase (below) (**D**). The red arrowhead indicates degenerated oocytes, and the red arrow indicates pycnotic oocytes

As previously stated, the Baicalin was found to help maintain the viability of granulosa cells cultured in vitro and their ability to produce steroid hormones, which is very important for normal ovarian function. Therefore, we further explored whether Baicalin could improve the ovarian function of aged female mice. Ten-month-old CD1 female mice were intragastrically administered with Baicalin (100 mg/kg) every other day, and a comparable control group was administered with saline. After 60 days of intragastric administration, vaginal cell smears were obtained daily for staining to monitor the estrous cycle of the mice. When compared with the aged control group, mice in the Baicalin treatment group had an increased estrus, a shorter interval, and an improved estrous cycle (Fig. 5A and B). Sectioning of the embedded mice ovaries and HE staining were performed after intragastric administration to analyze the development of the follicles in the ovary. There were more follicles at various stages in the ovaries of the aged mice treated with Baicalin than those in the control mice (Fig. 5C). Through superovulation, it was found that there was no significant difference in the morphology of the COCs between the control and Baicalin treatment groups (Fig. 5D). However, after removing the cumulus cells, the oocyte degeneration rate of the aged control group was significantly higher than that of the aged Baicalin group (86.36% versus 64.00%, respectively, *P* < 0.05) (Fig. 5D).

### MTOR pathway is involved in the regulation of granulosa cells and ovarian function by Baicalin

**Fig. 6:**
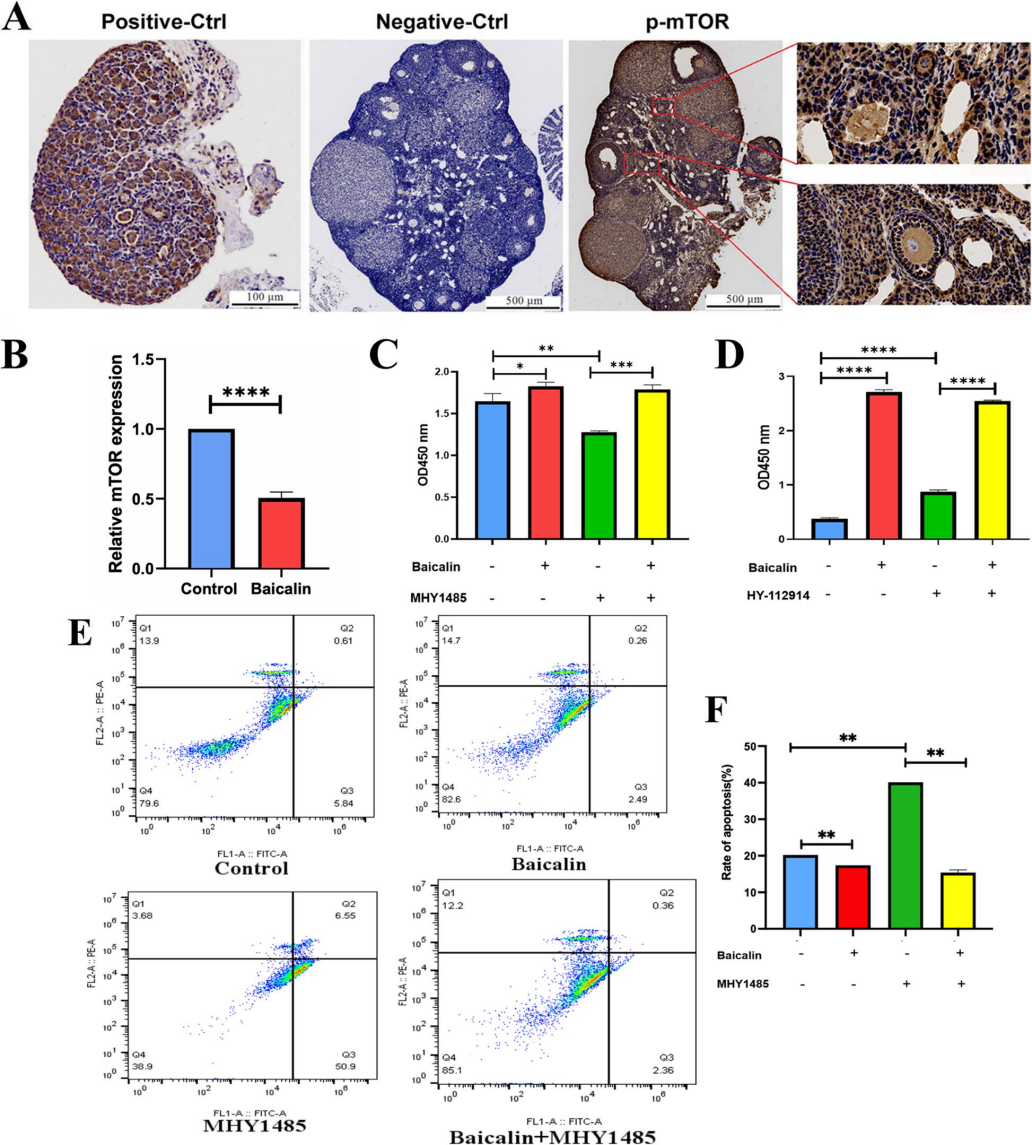
Baicalin may improve granulosa cell function and ovarian follicle development through the mTOR signaling pathway. The ovaries of the mice were routinely fixed, embedded, and sliced for p-mTOR immunohistochemical staining with a positive signal of brown (**A**). Positive control (Positive-Ctrl, left), Negative control (Negative-Ctrl, middle). Granulosa cells were starved for 24 h after conventional culture. Then the total RNA of the cells was extracted following treatment with Baicalin. The mRNA expression levels of the mTOR were detected using real-time PCR (**B**). Granulosa cells were starved for 24 h after conventional culture and then treated for 72 h as shown in the figure. The corresponding mTOR activator (MHY1485) and inhibitor (HY-112914) were then used, followed by cell viability detection using a CCK-8 cell viability detection kit (**C** & **D**). Flow cytometry was used to detect the apoptosis rate (**E** & **F**). * *P* < 0.05, ** *P* < 0.01, *** *P* < 0.001, and **** *P* < 0.0001

To further explore whether the mTOR signaling pathway was involved in the regulation of granulosa cell function and follicular development by Baicalin, the mTOR activator/inhibitor was used and immunohistochemical analysis was performed. The results showed that p-mTOR was expressed in the cytoplasm of granulosa cells and oocytes at all stages of follicle development in mice with a stronger positive expression in oocytes (Fig. 6A). Real-time PCR was used to detect the mRNA expression levels of mTOR, and the results showed that the Baicalin treatment significantly decreased the mRNA expression levels of the mTOR in the granulosa cells (*P* < 0.0001; Fig. 6B).

Primary granulosa cells were pretreated with MHY1485 (mTOR activator) for 30 min and then simultaneously treated with Baicalin for 72 h. The cell viability test results showed that MHY1485 significantly inhibited cell viability (*P* < 0.01), whereas Baicalin significantly reversed its inhibitory effect (*P* < 0.001; Fig. 6C) and that HY-112914 (mTOR inhibitor) significantly improved cell viability (*P* < 0.0001; Fig. 6D). The results of apoptosis detection by flow cytometry analysis showed that MHY1485 significantly promoted the apoptosis of granulosa cells cultured in vitro (*P* < 0.01), whereas Baicalin significantly inhibited the pro-apoptotic effects of MHY1485 (*P* < 0.01; Fig. 6E and F).

## Discussion

Previously, little was known about the specific effects of Baicalin on reproduction in female mammals, especially in relation to follicular development, and its underlying mechanisms. In this investigation, we conducted experiments to improve our understanding of these issues. We found that a low concentration of Baicalin helped to maintain a favorable growth state of granulosa cells when under in vitro culture conditions, inhibit cell apoptosis, reverse the damage of oxidative stress, and impact on the secretion of steroid hormones. By intragastrically administering Baicalin to aged mice, it was found that it could improve their estrous cycle and the quality of their oocytes. Further, we found that Baicalin may improve the granulosa cells and ovarian function through the mTOR signaling pathway.

This study is mainly to explore the effects of Baicalin on granulosa cell function and ovarian function. In terms of granulosa cell function, the main research target is human primary granulosa cells. In order to further explore the general applicability of Baicalin to the regulation of granulosa cell function, we also explored the effect of Baicalin on the growth and cell viability of the human granulosa cell line KGN in vitro.

The growth and development of follicles and the maturation of oocytes is closely related to the viability of the granulosa cells in the follicles [18]. The development of mammalian oocytes requires effective interactions of the granulosa cells in the follicles, as the proliferation of the granulosa cells supports and promotes normal follicle growth and development [19]. In this study, granulosa cells were cultured in vitro and treated with different concentrations of Baicalin to examine the effects. It was previously reported that low concentrations of Baicalin can reduce the apoptotic rate of bovine mammary epithelial cells (BMECs), while high concentrations can promote cell apoptosis [20]. These previous results were consistent with our findings that low concentrations of Baicalin were beneficial for the growth of granulosa cells cultured in vitro, while high concentrations of Baicalin were found to promote cell apoptosis.

We know that Baicalin taken by organisms under normal conditions is generally at a low concentration in the body under physiological conditions. For example, 10 μM in our research will be beneficial to the positive effects of Baicalin on the body or cells. Once Baicalin is higher than this physiological concentration, it may cause stress to the body or cells, resulting in toxic to the body or cell. It has been reported that autophagy is also one of the causes of direct cell death. Autophagy may degrade cells by changing the levels of key apoptosis regulators, thereby promoting cell apoptosis [21]. In our research, it was found that Baicalin may regulate the autophagy process through the mTOR signaling pathway. Therefore, high concentration of Baicalin may also trigger excessive autophagy, leading to autophagy death mechanism and causing cell toxicity.

The growth environment of granulosa cells in the ovarian follicles is influenced by aging and the simulation of the external environment, resulting in the excessive accumulation of ROS in the granulosa cells and oxidative stress, which ultimately leads to abnormal follicular growth and development. In this study, we constructed an oxidative stress model of granulosa cells induced by hydrogen peroxide and found that Baicalin could reverse this induced oxidative stress damage and play a role in cell protection. Several studies have previously reported that Baicalin can inhibit cell apoptosis and cell damage which can be caused by a variety of reasons in different systems and organs. In a rat model, Baicalin was found to alleviate the symptoms of acute injury and inhibit the apoptosis of liver and kidney tissues, thereby exerting a protective effect on these organs [22]. Baicalin was also found to inhibit the apoptosis of BMECs by reducing the generation of cellular ROS [20] and attenuated cell damage in the rat pheochromocytoma cell line (PC12) that was induced by hydrogen peroxide [23]. Arsenic trioxide is an environmental pollutant that can inhibit the viability of human umbilical vein endothelial cells (HUVECs) and promote cell apoptosis, while Baicalin can reverse the toxic effects of arsenic trioxide on HUVECs [24].

Pan et al. (2019) found that Baicalin could effectively inhibit the apoptosis of mouse myoblasts (C2C12), reverse the apoptosis of C2C12 cells induced by hydrogen peroxide, decrease the expression of Caspase 3 and Caspase 9, and reduce oxidative stress damage to skeletal muscles [25]. Baicalin pretreatments were also found to increase the viability of neuroblastoma cells (SH-SY5Y) and inhibit the apoptosis-inducing effects of hydrogen peroxide on neurocytes, which may be related to the upregulation of SIRT1 and the downregulation of Caspase 3 [26]. Furthermore, it was confirmed that Baicalin administration could significantly inhibit the expression of Caspase 3 in the ischemic hippocampus of gerbils, indicating that Baicalin has protective effects on the nervous system [27]. It has been confirmed that medium doses of Baicalin can inhibit the apoptosis of human placental choriocarcinoma JEG-3 cells, which may be related to the inhibited expression of apoptosis-related proteins (Caspase 3, Caspase 6, and Caspase 9) and the promoted expression of Bcl-2 and the X-linked inhibitor of the apoptosis protein [22]. These results were consistent with our findings that Baicalin promoted the expression of Bcl-2 protein in granulosa cells cultured in vitro and inhibited the expression of Bax and Caspase3.

The steroid-producing ability is an important function of ovarian granulosa cells, and an important inducing factor for the normal growth and development of oocytes and the proliferation and differentiation of granulosa cells. Estrogen is an important regulator of female reproductive function. The presence of estrogen can promote the growth of the endometrium to help prepare it to accept embryo implantation. Studies have confirmed that estrogen signaling in the body is closely related to epigenetic mechanisms. Post-translational histone modification, microRNA (miRNA) expression, and DNA methylation are regulated by estrogen signaling. Some estrogen signaling regulators also show certain chromatin modification activity [28]. Thus, estrogen plays an important role in both physiological and pathological processes in females. In the reproductive system, progesterone can make the proliferative endometrium transition from proliferation to secretion, which is beneficial to blastocyst implantation and essential for maintaining pregnancy. Moreover, progesterone is also associated with many other systemic diseases, including Alzheimer’s disease, cerebral edema, osteoporosis, and diabetic neuropathy [30]. Ma et al. (2009) found that the intragastric administration of Baicalin during pregnancy in mice could reduce the occurrence of embryonic death caused by bromocriptine intervention, which might be related to increased progesterone and reductions in embryonic death with Baicalin treatments [31]. Consistent with these results, this study has also found that Baicalin increased the secretion of estradiol and progesterone in the granulosa cells. Furthermore, by intragastrically administering Baicalin to aged mice, we found that it could improve their estrous cycle, which may be associated with the ability of Baicalin to increase hormone secretion in the granulosa cells.

At present, animal experiments have confirmed that Baicalin has an anti-abortion effect. When compared with in vivo conditions, the mouse embryo quality and blastocyst development rate are significantly decreased when cultured in vitro. The addition of Baicalin significantly improved the development ability of mouse embryos in vitro, which was associated with the effects of inhibiting apoptosis and reducing cellular stress by Baicalin [32]. Guo et al. (2019) found that Baicalin could inhibit the production of ROS, reduce oocyte apoptosis, and improve the maturity of porcine oocytes and subsequent embryonic development in vitro [33]. In the LPS-induced abortion model in mice, Baicalin exerted an anti-abortion effect by inhibiting the maternal-fetal immunity and expression of IL-10 and protecting the decidual cells [34]. Experimental studies in mice found that Baicalin might upregulate the expression of fucosyltransferase IV (FUT4) through the Wnt/β-catenin signaling pathway, thus significantly increasing the implantation rate of mouse embryos [37]. After 90 days of intragastric administration of Baicalin to the aged mice, we found that it significantly reduced the degeneration rate and improved the quality of their oocytes.

In view of the current research reports, Baicalin clearly has important anti-apoptotic and anti-oxidative stress effects, and some researchers have explored the action pathways of Baicalin. Sui et al. (2019) found that Baicalin could alleviate testicular tissue damage and cell apoptosis in mice caused by acute heat stress by blocking the Fas/FasL pathway [38]. Other studies have reported that Baicalin can exert anti-oxidative stress effects by activating nuclear factor erythroid 2-related factor 2 (Nrf2). In H9C2 cells, Baicalin inhibited cell viability decline and apoptosis by activating the Nrf2/heme oxygenase 1 (HO-1)-mediated HIF1α/BNIP3 pathway [39]. Baicalin also inhibited oxidative stress and inflammation by activating the Nrf2/HO-1 signaling pathway, leading to a significant improvement in lipopolysaccharide-induced acute lung injury [40]. The study of HK-2 proximal tubule epithelial cells from the human renal cortex found that Baicalin pretreatments could activate the downstream Nrf2 pathway to inhibit the cytotoxic effects of hydrogen peroxide on HK-2 [41]. It was reported that the mTOR pathway played an important role in follicular development, and that Baicalin could regulate the mTOR signaling pathway of human skin fibroblasts and lung cancer cells [42]. Our results showed that Baicalin treatments could affect the mTOR pathway, and its activation or inhibition could affect the actions of Baicalin on the granulosa cells.

mTOR is a serine/threonine kinase and an important cell regulator, which can regulate cell growth and proliferation in response to a variety of signals, and it plays an especially vital role in regulating autophagy [44]. mTOR is a highly evolved kinase whose catalytic core is composed of two different complexes, mTORC1 and mTORC2 [45]. Yu et al. (2021) found that Qingfei oral liquid (QF) could participate in the regulation of autophagy through the mTOR signaling pathway to reduce the inflammation caused by respiratory syncytial virus (RSV) infection in asthmatic mice [46]. The Castets research team used rapamycin to inhibit mTORC1 and found that mTORC1 and mTORC2 played key roles in controlling autophagy in the skeletal muscles of young and old people [47]. Their study also found that the mTORC1-autophagy axis and mTORC1-Akt axis were essential for the recovery of coordination after denervation [48]. Similarly, Xu et al. (2021) reported that Orexin A (OXA) could inhibit over-active autophagy in the nervous system by modulating the OX1R-mediated MAPK/ERK/mTOR pathway, thereby exerting neuroprotective effects in vivo and in vitro [49]. These findings indicated that mTOR was involved in the regulation of autophagy in multiple systems in the body.

Through immunohistochemical experiments, we found that p-mTOR was expressed in the cytoplasm of the granulosa cells and oocytes of ovarian follicles at all stages of development in mice, with a stronger expression level in oocytes, indicating that mTOR was involved in regulating the development of ovarian follicles. It has also previously been found that knockouts of the autophagy-inducing gene Atg7 could lead to low fertility in female mice, as autophagy can protect the oocytes in the neonatal ovaries from the excessive loss of follicles caused by apoptosis under starvation conditions [50]. It was reported that pretreatments with rapamycin (mTOR inhibitor) before vitrification of the ovarian tissue could increase the survival rates of the ovarian tissues after freezing and thawing, and this was also considered to be an effect on the preservation of follicle reserves and the promotion of ovarian survival during cryopreservation and the transplantation of ovarian tissues [51]. In addition, mTOR inhibitors were also found to protect the ovarian reserves in mice treated with cyclophosphamide, with positive effects on the maintenance of the number of primordial follicles, serum anti-Müllerian hormone levels, and fertility [52]. Yorino et al. (2020) also reported that rapamycin could help to maintain the follicle reserves in mice [53]. Therefore, we speculated that Baicalin had a regulatory effect on the development of ovaries and follicles through the mTOR signaling pathway in mice. In addition, according to our research results, Baicalin may also regulate autophagy through the mTOR pathway, thereby affecting the development of ovarian follicles.

Autophagy regulation and autophagy disorders are the basis of many pathological diseases targeted by traditional Chinese medicines, and it has been found that an increasing number of Chinese herbal medicines act as autophagy regulators. This autophagy regulation effect is consistent with traditional medication methods and plays a beneficial role in the treatment of many diseases [54]. Our study found that Baicalin could significantly inhibit the mRNA expression levels of mTOR, suggesting that Baicalin might improve the functions of granulosa cells and the development of follicles by regulating the mTOR pathway. Further studies found that mTOR activator treatments could significantly downregulate granulosa cell viability, while the mTOR inhibitor could significantly increase granulosa cell viability. Experiments on apoptosis showed that Baicalin could restore the apoptosis-promoting effects of the mTOR activator, which further illustrated that this may be how Baicalin improves the function of follicular granulosa cells and the development of follicles.

## Conclusions

The results of this study indicate that Baicalin helps to increase the viability of granulosa cells cultured in vitro, reduce the damage due to oxidative stress in these cells, and promote their secretion of estradiol and progesterone. In vivo experiments in aged mice showed that Baicalin helps to improve their estrous cycle and the quality of their oocytes. It was found that the effect of Baicalin can be achieved at least in part by regulating the mTOR signaling pathway. Further in-depth studies on the role of Baicalin in the development of ovarian follicles and its mechanisms will likely provide new ideas and methods to improve the treatment of ovarian dysfunction and delay the process of ovarian aging.

## Supplementary Information

**Additional file 1: Supplementary Figure S1.** Effects of high concentrations of Baicalin on cultured KGN and primary granulosa cells in vitro. After 24 h in the starvation culture, different concentrations of Baicalin (0, 100, 150, and 200 μM) were added. The processing start time was set to 0 h, and then observations and photography were performed after 48, 72, and 96 h. The left picture shows the KGN granulosa cell line (A), and the right picture shows the primary granulosa cells (B). The control group in this figure is the same as the control group in Fig. 1. Scale bar = 50 μm.

**Additional file 2: Supplementary Table 1**. Primers used in this study.

## Data Availability

Data and related analysis are available from the corresponding authors on reasonable request.

## Abbreviations

COC: Cumulus–oocyte complex
FUT4: Fucosyltransferase IV
hCG: Human chorionic gonadotropin
HE: Hematoxylin-eosin
HO-1: Heme oxygenase 1
HRP: Horseradish peroxidase
HUVECs: Human umbilical vein endothelial cells
mTOR: Mammalian target of rapamycin
Nrf2: Nuclear factor erythroid 2-related factor 2
PBS: Phosphate-buffered saline
PMSG: Pregnant mare serum gonadotropin
