# Supplementary material for: Baicalin improves the functions of granulosa cells and the ovary in aged mice through the mTOR signaling pathway
Source: J Ovarian Res. 2022 Mar 17;15:34. doi: 10.1186/s13048-022-00965-7 (PMC8932175; doi:10.1186/s13048-022-00965-7)
Supplement: Supplementary file 1 — Additional file 1: Supplementary Figure S1. Effects of high concentrations of Baicalin on cultured KGN and primary granulosa cells in vitro. After 24 h in the starvation culture, different concentrations of Baicalin (0, 100, 150, and 200 μM) were added. The processing start time was set to 0 h, and then observations and photography were performed after 48, 72, and 96 h. The left picture shows the KGN granulosa cell line (A), and the right picture shows the primary granulosa cells (B). The control group in this figure is the same as the control group in Fig. 1. Scale bar = 50 μm. [file 13048_2022_965_MOESM1_ESM.docx]

**Supplementary Figure**


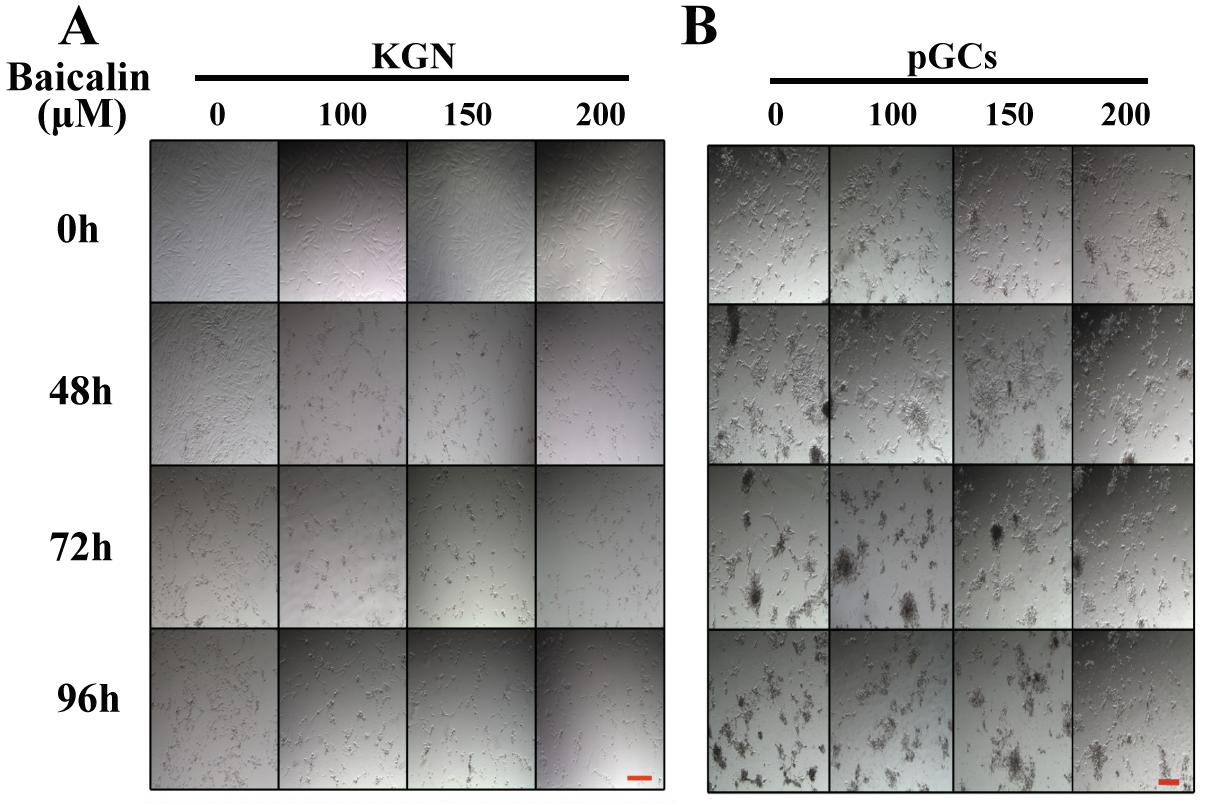


**Supplementary Figure S1. Effects of high concentrations of Baicalin on cultured KGN and primary granulosa cells *in vitro*.** After 24 h in the starvation culture, different concentrations of Baicalin (0, 100, 150, and 200 μM) were added. The processing start time was set to 0 h, and then observations and photography were performed after 48, 72, and 96 h. The left picture shows the KGN granulosa cell line (A), and the right picture shows the primary granulosa cells (B). The control group in this figure is the same as the control group in Figure 1. Scale bar = 50 μm.
