# Supplementary material for: Baicalin improves the functions of granulosa cells and the ovary in aged mice through the mTOR signaling pathway
Source: J Ovarian Res. 2022 Mar 17;15:34. doi: 10.1186/s13048-022-00965-7 (PMC8932175; doi:10.1186/s13048-022-00965-7)
Supplement: Supplementary file 2 — Additional file 2: Supplementary Table 1. Primers used in this study. [file 13048_2022_965_MOESM2_ESM.docx]

**Supplementary Table 1. Primers used in this study**

| **Gene names** | **Forward Primer** | **Reverse Primer** |
| --- | --- | --- |
| *GAPDH* | 5'-ATGGAAATCCCATCACCATCTT-3' | 5'- CGCCCCACTTGATTTTGG-3' |
| *BAX* | 5'-TCAGGATGCGTCCACCAAGAAG-3' | 5'TGTGTCCACGGCGGCAATCATC-3' |
| *BCL-2* | 5'-ATCGCCCTGTGGATGACTGAGT-3' | 5'GCCAGGAGAAATCAAACAGAGGC-3' |
| *stAR* | 5'-TACGTGGCTACTCAGCATCGAC-3' | 5'-TCAACACCTGGCTTCAGAGGCA-3' |
| *P450arom* | 5'-GACGCAGGATTTCCACAGAAGAG-3' | 5'-ATGGTGTCAGGAGCTGCGATCA-3' |
| Caspase 3 | 5'-GGAAGCGAATCAATGGACTCTGG-3' | 5'-GCATCGACATCTGTACCAGACC-3' |
| *MTOR* | 5'-AGCATCGGATGCTTAGGAGTGG-3' | 5'-CAGCCAGTCATCTTTGGAGACC-3' |
